# Robotic device for transcranial focussed ultrasound applications in small animal models

**DOI:** 10.1002/rcs.2447

**Published:** 2022-08-12

**Authors:** Anastasia Antoniou, Marinos Giannakou, Elena Georgiou, Kleopas A. Kleopa, Christakis Damianou

**Affiliations:** ^1^ Department of Electrical Engineering, Computer Engineering, and Informatics Cyprus University of Technology Limassol Cyprus; ^2^ R&D, Medsonic Ltd Limassol Cyprus; ^3^ Department of Neuroscience The Cyprus Institute of Neurology and Genetics Nicosia Cyprus

**Keywords:** BBB disruption, focussed ultrasound, mice, MRI compatible, robotic device, transcranial

## Abstract

**Background:**

Focussed Ultrasound (FUS) combined with microbubbles (MBs) was proven a promising modality for non‐invasive blood brain barrier disruption (BBBD). Herein, two devices for FUS‐mediated BBBD in rodents are presented.

**Methods:**

A two‐axes robotic device was manufactured for navigating a single element FUS transducer of 1 MHz relative to the brain of rodents. A second more compact device featuring a single motorized vertical axis was also developed. Their performance was assessed in terms of motion accuracy, MRI compatibility and trans‐skull BBBD in wild type mice using MBs in synergy with pulsed FUS.

**Results:**

Successful BBBD was evidenced by the Evans Blue dye method, as well as by Fibronectin and Fibrinogen immunostaining. BBB permeability was enhanced when the applied acoustic intensity was increased.

**Conclusions:**

The proposed devices constitute a cost‐effective and ergonomic solution for FUS‐mediated BBBD in small animal models. Further experimentation is needed to examine the repeatability of results and optimise the therapeutic protocol.

## 1 INTRODUCTION

Penetration of the blood‐brain barrier (BBB) to deliver medication into the brain is a subject that has aroused the interest of many research groups. The techniques available so far are not very effective. The BBB, which is the body's defence against toxic substances, also provides resistance to the supply of therapeutic agents. Therefore, the provision of medication to the brain is a main problem to overcome. In this regard, focussed ultrasound (FUS) seems to be an alternative completely non‐invasive method that can enhance treatment against neurodegenerative diseases.^1^

It has been shown that opening of the BBB can be achieved with the use of therapeutic ultrasound and the administration of microbubbles (MBs).^2^ This process is reversible, thus maintaining the ability of the brain to stay protected against harmful substances. Specifically, application of pulsed FUS induces various mechanical phenomena in tissue, which in synergy with MBs, loosen the endothelial cell connections allowing medication to reach the brain.^3^ This method is targeted since the ultrasonic energy is focussed at a specific area of the brain, thus reducing the risk for complications from the process.^4^ The relaxation of the endothelial ligaments is completely reversible, with complete recovery occurring within a few hours after the treatment.^5^ Since low intensity FUS is used, the temperature remains at safe levels.

The application of this method for disrupting the BBB has been tested in various animal models, but mostly evaluated in mice and rats.^6^, ^7^, ^8^ Due to their small size, mice are easier to handle and allow the use of more economical infrastructure, compared to larger animals. However, their small size also appeared to be a challenge in terms of accurate targeting in the laboratory environment, where MRI feedback is not available. For this reason, various experimental devices have been used by several research groups involved in the field to facilitate studies in small animal models.

The team of Konofagou did remarkable work in the field using a 3‐axis robotic system (Velmex Inc., Lachine, QC, Canada).^9^, ^10^, ^11^, ^12^, ^13^ The FUS transducer was attached to the positioning system, as well as to a water‐filled cone. Another water tank featuring an acoustic opening at the bottom was used,^9^, ^10^, ^11^, ^12^ and coupled to the mouse head using ultrasound gel.^13^ The water tank was stable allowing the transducer as integrated with the water‐filled cone to move inside the tank relative to the target, without affecting the coupling with the mouse head.

A manual mounting system was proposed by the team of Hynynen.^5^ The mouse was placed in the supine position above a water container. The transducer was positioned in the container under the mouse head and acoustic coupling was achieved using a bag filled with water. Similar experimental setups as the ones described above with some modifications were used in relevant studies.^14^, ^15^, ^16^, ^17^

There are also systems available in the market that were developed for research activities. An example is the PK50 system offered by the FUS Instruments company (Toronto, Canada). The system has 3 degrees of freedom (DOF) for transducer positioning.^18^ This company also offers another mounting device with 3 DOF, which approaches the target from the bottom (LP100, FUS Instruments, Toronto, Canada).^18^ Another company that offers robotic devices for research purposes is Verasonics (Kirkland WA, USA).^19^ The company owns a robotic system with 2 DOF, where ultrasonic coupling is achieved using a water filled bladder. The guidance of the system is achieved with diagnostic ultrasound.^19^

Image guided therapy manufactures robotic systems compatible with MRI. This company offers 2 different robotic systems^20^ featuring 5 DOF. These systems are intended for various therapeutic ultrasound applications. However, they are complex and thus not ergonomic, especially for small animal experiments.^20^

The company Sonovol focuses on imaging modalities for preclinical applications,^21^ but it also offers a preclinical device for FUS applications guided by three‐dimensional ultrasound combined with acoustic angiography. The system was designed to assist research with therapeutic ultrasound, given that fusion of ultrasound imaging and angiography can be beneficial for guiding BBB disruption (BBBD). Notably, the system offers a wide field of view combining the two imaging modalities.

Robotic‐assistance was introduced in many studies to improve the accuracy of ultrasonic targeting.^22^, ^23^, ^24^, ^25^, ^26^, ^27^ As an example, Kujawska et al.^25^, ^26^ developed a computer‐controlled robotic system with 4 DOF for FUS ablation preclinical studies. The 4 DOF positioner is attached on a water‐filled tank to maneuver a dedicated platform that carries the target relative to the FUS transducer, which is fixed coaxially with an ultrasound imaging probe on the bottom of the tank facing towards the underside of the target.

There is an increasing demand for preclinical robotic devices, as various FUS applications are continuously being developed and should be investigated to demonstrate the accuracy and repeatability needed for their clinical translation. Preclinical devices are the most cost‐effective solution because medical certification is not necessary. Although numerous devices with different functionalities have been developed and tested so far, more simplistic and ergonomic devices dedicated for small experimental animals would be of great usefulness in accelerating research in the field.

In this study, we propose two systems dedicated to manoeuvering a single element FUS transducer for preclinical research in small animal models. The first system had the ability to manoeuver the transducer in two dimensions. The operation of the system is simplistic since all the moving parts are placed in a single water tank that includes an acoustic window on the top. A target supporting platform was specially designed to securely position rodents above the ultrasonic source.

A second system was built to simplify targeting given the very small size of the mouse head and offer improved ergonomics. In this version, the mouse is placed in the more stable prone position on a flat platform, with the transducer reaching the head with a top to bottom approach. In fact, the transducer is located inside a cone that is acoustically coupled to the mouse head using ultrasound gel. With this design, the administration of anaesthesia is more flexible.

Both devices were made MRI compatible. Even though the two devices were primarily developed for laboratory use, MRI compatibility is important since it allows for treatment planning and accurate targeting in the MRI setting, as well as confirmation of BBBD by contrast agent enhanced imaging directly after treatment.

The proposed devices will provide the researchers with means to perform research on FUS applications in small animals. The two devices were engineered in a way that ensures ease of use, with adjustment tools to suit the different species. Especially for very small animals such as mice, the accuracy benefits of the proposed experimental setup are of high importance. Overall, the proposed systems are easy to make at an affordable price and were developed based on the knowledge gained from our previously introduced robotic systems.^28^, ^29^, ^30^, ^31^, ^32^, ^33^

## 2 MATERIALS AND METHODS

### 2.1 Focussed ultrasound setup

A custom‐made FUS transducer was manufactured in‐house using a single piezoceramic element (Piezo Hannas Tech Co. Ltd, Wuhan, China), with a radius of curvature of 80 mm, an active diameter of 50 mm, and an operating frequency of 1 MHz. A dedicated housing was 3D printed using Acrylonitrile styrene acrylate (ASA) material on a STRATASYS (F270, Eden Prairie, Minnesota, USA) printer having a circle‐shaped cavity, wherein the element was soldered. An electric circuit was created and encapsulated with epoxy, which serves as electric isolator and simultaneously as a backing material preventing excessive vibration of the element and improving the acoustic performance of the transducer. The acoustic efficiency of the transducer was experimentally determined at 33% by the radiation force balance method.^34^ Note that the selection of the various transducer components was based on MR‐compatibility.

The transducer is tuned to an RF amplifier (AG series, T&G Power conversion Inc., Rochester, NY) and its actuation is controlled via an in house developed software, which allows selection between continuous and pulsed ultrasound sonication. There is also the possibility to set the sonication parameters, such as the electric power, sonication duration, frequency, and duty cycle.

### 2.2 Positioning devices

#### 2.2.1 Robotic positioning device V1

**FIGURE 1:**
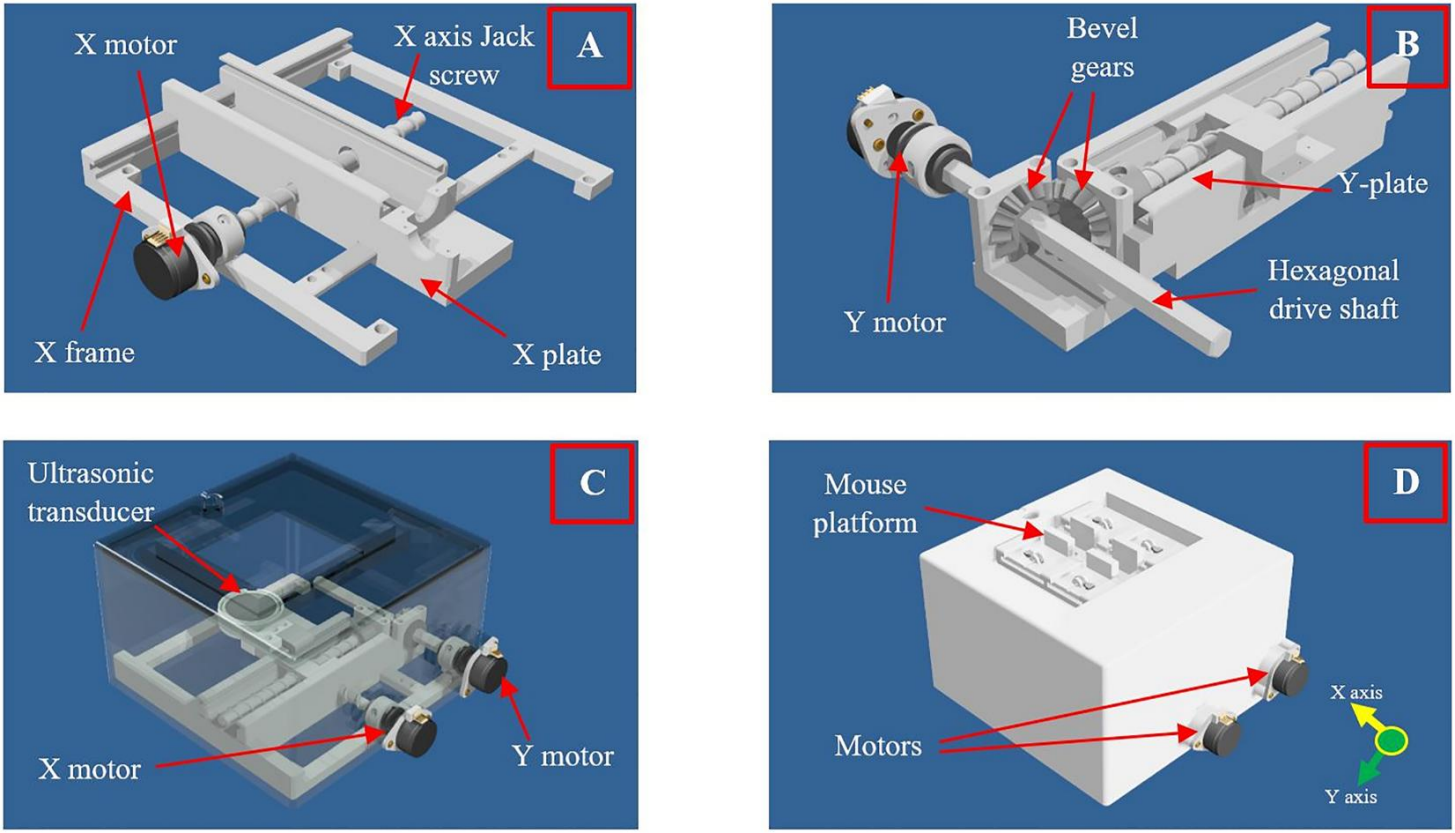
CAD drawings of the (A) X‐stage, (B) Y‐stage, (C) positioning device with transparent enclosure, and (D) positioning device

A 2 DOF motorized device was manufactured using a 3D printing machine (FDM 270, Stratasys, Minnesota, USA). Figure 1 shows computer‐aided design (CAD) drawings of the device revealing its components and how they are assembled. The various parts were produced using the fused disposition modelling (FDM) technology with ASA thermoplastic. The positioning mechanism maneuvers the proposed transducer in the *X* and *Y* linear axes, with a motion range of 60 and 130 mm, respectively. Specifically, the rotational motion of two piezoelectric motors (USR30‐S3; Shinsei Kogyo Corp., Tokyo, Japan) located outside the water enclosure is converted into linear motion via complex mechanisms located inside the enclosure, as shown in Figure 1.

The *X* axis angular motion is converted into linear motion by a Jack screw mechanism. The motor rotates the Jack screw that is linked with the *X*‐plate (Figure 1A). The rotation of the Jack screw in turn causes the *X* plate to move forward (upon counterclockwise rotation) or back (upon clockwise rotation) along dedicated guides of the *X*‐frame, which has also a supportive role increasing structural rigidity. The pitch of the Jack screw is 1.44 cm, meaning that for each complete rotation, the *X* stage moves 1.44 cm.

The *Y* axis mechanism involves additional moving parts since the motion has to be delivered at a 90° angle (Figure 1B). The motor was placed outside the water container and was connected to a hexagonal drive shaft for transferring the motion to the interior parts. Bevel gears were coupled to the shaft transferring the motion at 90° (along the *Y* axis). Bevel gears refers to a type of gears with conically shaped teeth that transmit motion at an angle. The gear rotates the *Y* axis jackscrew, thus converting rotational motion into linear motion of the *Y* plate. The angular to linear motion ratio of the *X* and *Y* axes is equal, thus establishing uniformity.

The entire mechanism operates within the water container (Figure 1C), which is sealed by a cover (Figure 1D) having a square acoustic opening on the top. A platform with adjustable plates is fixed to the opening to secure the mouse above the FUS transducer.

#### 2.2.2 Robotic positioning device V2

**FIGURE 2:**
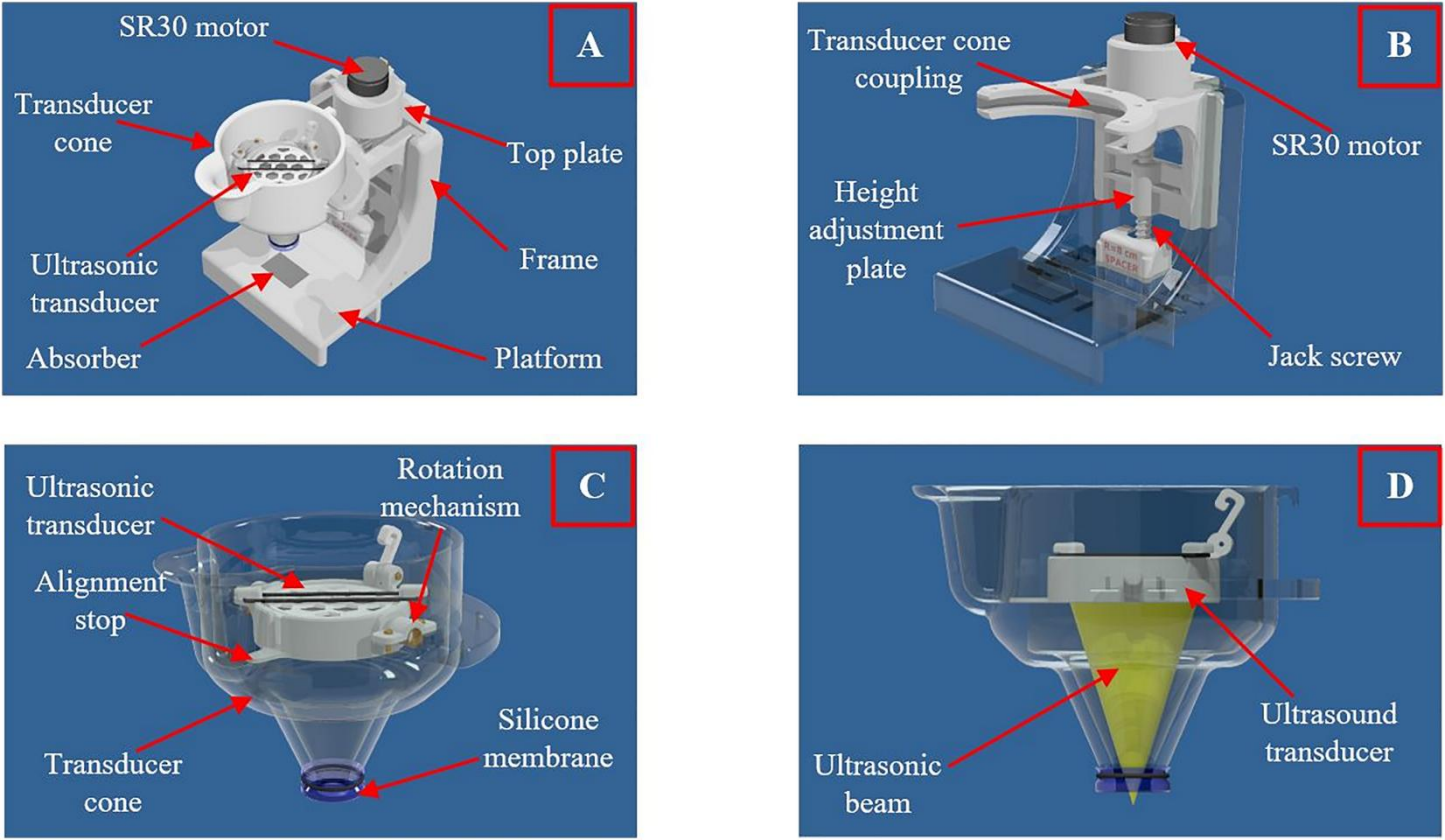
CAD drawings of the (A) robotic positioning device V2, (B) height adjustment mechanism, (C) transducer cone, and (D) transducer cone showing the ultrasonic beam

The second version of the device is shown in Figure 2A and was developed to achieve more efficient ultrasonic delivery in the mouse brain using a top to bottom approach. The main advantage of this approach is the ability to visually confirm proper coupling with the mouse head. Furthermore, this device was made smaller in size, and hence, it is lighter and easier to transport. Another essential benefit of this version is that intravenous injections and anaesthesia administration can be performed without removing the mouse from the device. For these reasons, it is considered more ideal for small animal experiments.

The device was manufactured on a polyjet 3D printing machine (Object30 pro, Stratasys, Minnesota, USA) using resin, which is cured when exposed to ultraviolet radiation. This technology offers high resolution, thus enabling the production of dimensionally accurate parts. The surface finish is also superior compared to the FDM technology where the layer lines are more visible.

This version of the device includes a flat platform where the mouse is positioned. Notably, an absorber was embedded in the centre of the platform for minimising ultrasound reflections. This platform is connected to a frame that includes linear guides for height adjustment via a moving plate (Figure 2B). The height adjustment plate carries a conical holder, which was designed to accommodate the FUS transducer (transducer cone in Figure 2C).

The height adjustment plate is operated in conjunction with a Jack screw having its first side attached to the platform and its second side connected to the top plate. The jackscrew is rotated by an ultrasonic motor (USR30, Shinsei, Tokyo, Japan) inducing vertical motion of the height adjustment plate so that the transducer cone can be fixed on targets of different size. Notably, its bottom part is securely sealed with a thin silicone membrane that is held by an O‐ring (Figure 2C). Upon operation, this cone is filled with degassed‐deionised water and is coupled to the target using ultrasound gel for proper ultrasonic transmission.

The transducer was mounted on the upper section of the cone using a special mechanism that enables its manual angulation. Angulation of the transducer is limited by a stop, thus ensuring the alignment of the ultrasonic beam with the acoustic opening (Figure 2D). This mechanism allows for easy removal of the air that is usually trapped on the transducer element during filling of the cone with water.

### 2.3 Power field assessment

The axial and radial power field of the designed transducer operating at its fundamental frequency of 1 MHz was evaluated by FUS field scanning with a hydrophone. A dedicated plastic holder was utilised to accommodate the designed transducer and the needle hydrophone (NH0500, Precision Acoustic, Dorset, UK) in an acrylic tank filled with degassed, deionised water. The transducer was precisely moved along the axial and radial directions by a system of stepping motors (VXM, Velmex Inc, Bloomfield, NY, USA) while the hydrophone was aligned to the beam axis to record the pressure waves at increasing distance from the transducer's surface. The hydrophone signal was displayed on a digital oscilloscope (TDS 2012, Tektronix, Inc., 14150 SW Karl Braun Drive, United States) and the peak to peak voltage recordings were collected. In total, 65 measurements were acquired with 2 mm intervals, in the range of 3–16 cm from the transducer's surface. At the estimated focal distance, 80 measurements were acquired in radial direction with 0.1 mm intervals. A voltage of 50 mV was applied in each case.

### 2.4 Motion accuracy assessment

The accuracy and repeatability of robotic motion for the two versions of the robot was assessed following a calliper‐based method as previously detailed in the literature.^35^ Briefly, motion steps of 1, 5, and 10 mm were commanded through the motion commands of the relevant software and compared with the actual displacements as measured with a high‐precision digital calliper. Additionally, the speed of motion in each axis was estimated by the activation time of the motion actuators, which is provided by the controlling software and equals to the time needed for the stage to cover the commanded step.

### 2.5 MRI compatibility assessment

The developed robotic devices were then evaluated in terms of proper operation in the MRI environment. Evaluation was carried out in a 1.5 T MRI scanner. The SNR served as the main tool for assessing the compatibility of the transducer with the scanner.

Imaging of an agar‐based tissue mimicking phantom (6% weight per volume agar; Merck KGaA, EMD Millipore Corporation, Darmstadt, Germany) was performed using the spoiled gradient recalled echo (SPGR) sequence with the following parameters: repetition time (TR) = 23 ms, echo time (TE) = 16 ms, flip angle (FA) = 35°, echo train length (ETL) = 1, pixel bandwidth (PB) = 45 Hz/pixel, field of view (FOV) = 280 × 280 × 10 mm^3^, matrix = 128 × 128, number of excitations (NEXs) = 2, and acquisition time/slice = 7 s.

The following activation states of the positioning mechanism were tested: motor/encoder cable not connected, motor/encoder cable connected, electronic control system energised but no motion command initiated (referred to as: DC ON), and motion command initiated (referred to as: motor moving). Regarding the FUS system, the following states were tested: RF cable not connected, RF cable connected, amplifier energised (zero power applied), and ultrasonic power applied. Electrical power values of 50–200 W were tested. In each case, the SNR was determined using the following formula^36^:

(1) SNR=SItargetσnoise

where the numerator is the mean signal intensity of a preselected target ROI while the denominator represents the standard deviation from a ROI placed in the air (noise).

### 2.6 Feasibility study in mice

Feasibility experiments were conducted in wild type (WT) mice (1‐month old, body weight 10–12 g) in collaboration with the Cyprus Institute of Neurology and Genetics to obtain proof of concept for the first version of the device. All the experimental procedures were approved by the Cyprus Veterinary Service under the protocol number CY/EXP/PR.L05/2021.

Initially, the transducer's location was adjusted to coincide with the circle‐shaped opening of the mouse holder (where the mouse head is fixed) through the motion commands of the interfaced software. The mouse head was shaved using hair removal cream. The mouse was then anaesthetized with isoflurane (Chanelle Pharm, I‐so‐vet®, Loughrea, Co Galway, Ireland) following administration of 10 or 20 μL of SonoVue MBs (Bracco Imaging, Turin, Italy) intravenously through the tail vein with a 30G syringe. Once the mouse was sufficiently anaesthetized, it was mounted on the device above the FUS transducer in the supine position and immobilised by properly adjusting the holder's handles. The container was filled with degassed‐deionised water up to the mouse head to ensure efficient ultrasonic coupling. It is essential to mention that before fixing the mouse to the holder, the transducer was energised enabling visual localization of the beam at the water surface, thus providing an additional reference for mouse positioning. Each mouse received a single sonication using FUS pulses of 10 ms length, applied at a repetition frequency of 1 Hz, for a total duration of 60 s using electrical power of 20 or 30 W.

In total, 6 mice were included in the study. Four (4) mice were treated using MBs‐enhanced FUS. The Evans Blue (EB) dye method was used to assess the success of BBBD. Specifically, 5 μL/g of body weight of a 4% EB stain solution (Sigma, St. Louis, MO, USA) was injected intravenously into each mouse immediately after sonication; 30 min before they were sacrificed. One mouse received EB only and another mouse served as the control mouse and received no treatment or EB.

All mice were sacrificed approximately 30 min after the sonication or/and EB administration. Slides containing brain sections were directly visualised using a Nikon eclipse‐Nἱ (Tokyo, Japan) fluorescence microscope to examine the EB extravasation. Furthermore, cryosections from brain were immunostained for fibronectin (DAKO, Glostrup, Denmark, 1:100) and FITC‐labelled polyclonal fibrinogen antibody (DAKO, 1:500) to assess the protein leakage into the parenchyma. DAPI staining (Sigma‐Aldrich, St. Louis, MO, USA) was used for nuclear localization (blue).

## 3 RESULTS

### 3.1 Power field assessment

Ultrasonic pressure field characterisation was performed using a hydrophone. The voltage recordings show a maximum pressure at 7.5 cm indicating that the actual focal spot is slightly shifted towards the transducer's surface. The axial pressure profile follows a Gaussian distribution with a full width half maximum (FWHM) of about 10 mm around the focus location (half pressure length). Accordingly, the radial pressure profile at the estimated focal distance of 7.5 cm also follows a Gaussian distribution around the central axis, which is characterised by a FWHM of about 4 mm (half pressure width). These measurements provide a good indication of the size of the focal spot.

### 3.2 Motion accuracy assessment

The results on motion accuracy as obtained by the calliper based method are summarised in Table 1, which lists the range of the measured actual displacements and the corresponding mean error for each axis direction and each commanded step. Note that the motion error decreases with increasing motion step, with a maximum mean positioning error of 0.080 ± 0.027 mm and 0.077 ± 0.026 mm for the first and second versions of the robot, respectively. Accordingly, the speed of motion was estimated at 9.90 ± 0.12 mm/s and 11.07 ± 0.17 mm/s in the *X* and *Y* directions, respectively. Regarding the second version of the robot, the Z‐stage was found to move with a speed of 8.65 ± 0.08 mm/s.

**TABLE 1:** The range of actual displacements as measured by the digital calliper at commanded motion steps of 1, 5, and 10 mm in each axis direction of the two robotic devices (version I and II), and the corresponding mean motion error and standard deviation

Version I	Commanded step (mm)	Range (mm)	Mean error ± SD forward (mm)	Mean error ± SD reverse (mm)
**X**	1	0.9–1.09	0.061 ± 0.031	0.064 ± 0.025
	5	4.9–5.06	0.046 ± 0.021	0.048 ± 0.022
	10	9.97–10.03	0.039 ± 0.010	0.036 ± 0.012

	Commanded step (mm)	Range (mm)	Mean error ± SD right (mm)	Mean error ± SD left (mm)
**Y**	1	0.88–1.1	0.08 ± 0.027	0.076 ± 0.032
	5	4.88–5.07	0.057 ± 0.029	0.051 ± 0.023
	10	9.95–10.04	0.023 ± 0.020	0.025 ± 0.016

Version II	Commanded step (mm)	Range (mm)	Mean error ± SD upward (mm)	Mean error ± SD downward (mm)
**Z**	1	0.86–1.1	0.073 ± 0.038	0.077 ± 0.026
	5	4.9–5.05	0.042 ± 0.026	0.05 ± 0.03
	10	9.92–10.03	0.028 ± 0.02	0.033 ± 0.024

### 3.3 MRI compatibility assessment

**FIGURE 3:**
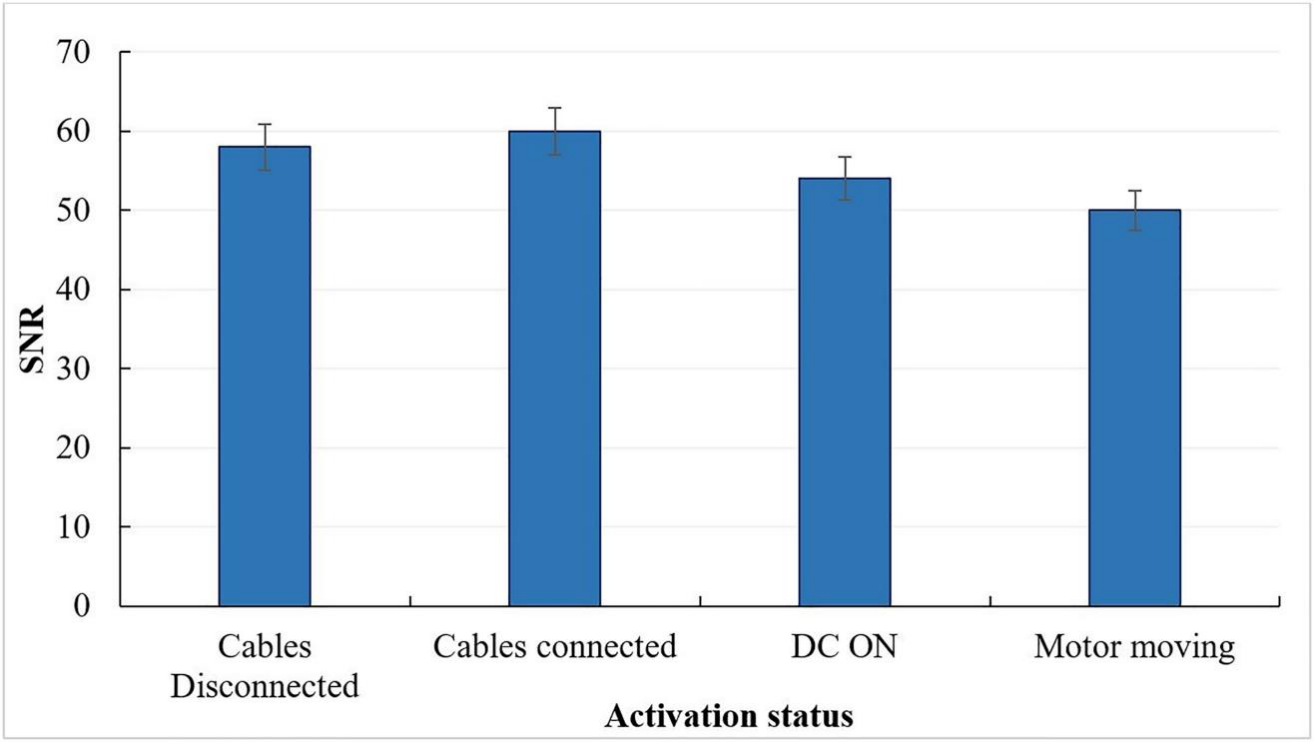
Bar chart of the SNR of SPGR images of an agar phantom acquired for different activation states of the robotic device (Cables Disconnected, Cables Connected, DC ON, and Motor moving). Error bars represent the standard deviation of the mean

**FIGURE 4:**
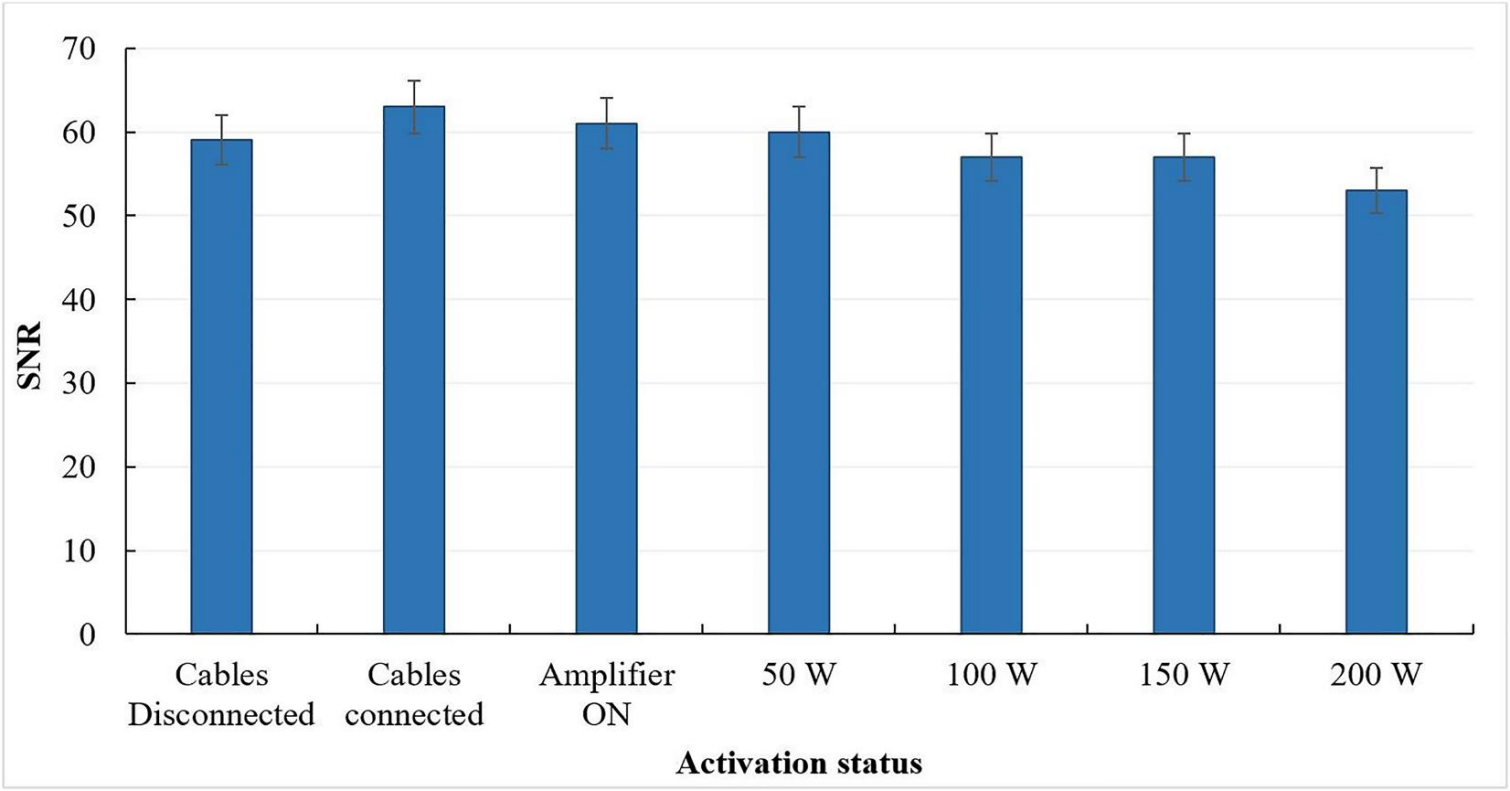
Bar chart of the SNR of SPGR images of an agar phantom acquired for different activation states of the FUS transducer (Cables Disconnected, Cables Connected, Amplifier ON, and power set at 50, 100, 150, and 200 W). Error bars represent the standard deviation of the mean

The bar charts of Figures 3 and 4 reveal how the SNR of SPGR images of the phantom is affected by changing the activation status of the system. The bar chart of Figure 3 shows the SNR estimations with the positioning mechanism being at different activation states. The greatest SNR reduction occurred when the ultrasonic motor was moving during image acquisition. The corresponding results for the FUS transducer are shown in Figure 4, which shows a gradual SNR reduction with increasing electric power from 50 to 200 W, most probably owing to the increasing target vibration. The MR compatibility was tested for version 1, which represents the worst case since it accommodates two motors.

### 3.4 Feasibility study in mice

**FIGURE 5:**
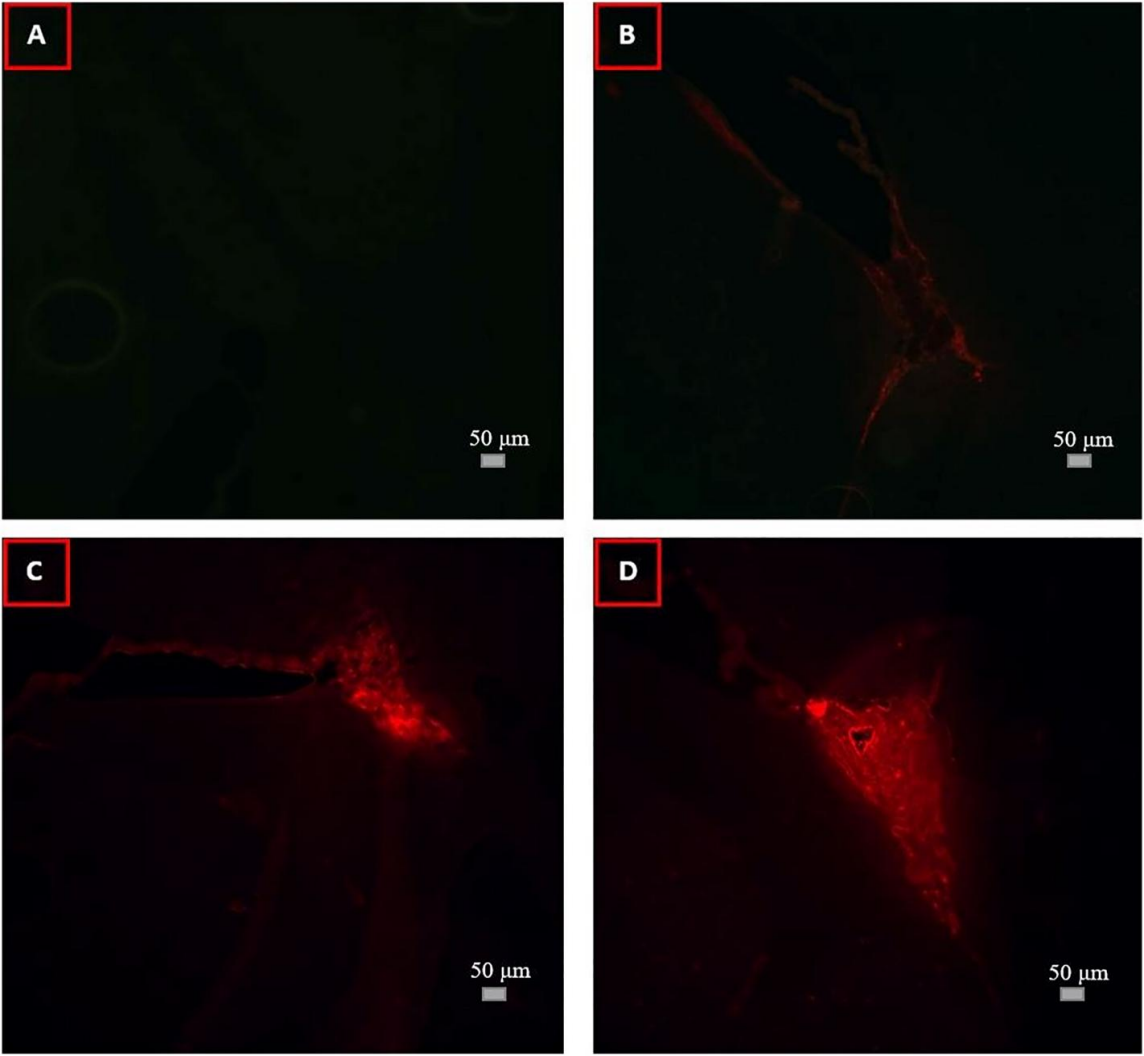
Fluorescence images of unstained brain sections at the level of the lateral ventricles taken from (A) a control mouse, (B) a mouse injected with EB only, and mice treated using (C) 20 W and 10 μL MBs, and (D) 30 W and 10 μL MBs (Scale bar: 50 μm)

BBB opening was evidenced in all cases (4/4). Representative microscopy photos of EB extravasation in the brain parenchyma adjacent to the lateral ventricles are shown in Figure 5. No leakage was observed in the brain parenchyma of the control mouse (Figure 5A) and the mouse injected with EB only (Figure 5B). EB leakage is clearly visible in red colour in mice treated with FUS in synergy with MBs (Figure 5C,D). Note that the mouse treated with higher acoustic power showed higher levels of EB dye in the brain tissue covering a larger area.

**FIGURE 6:**
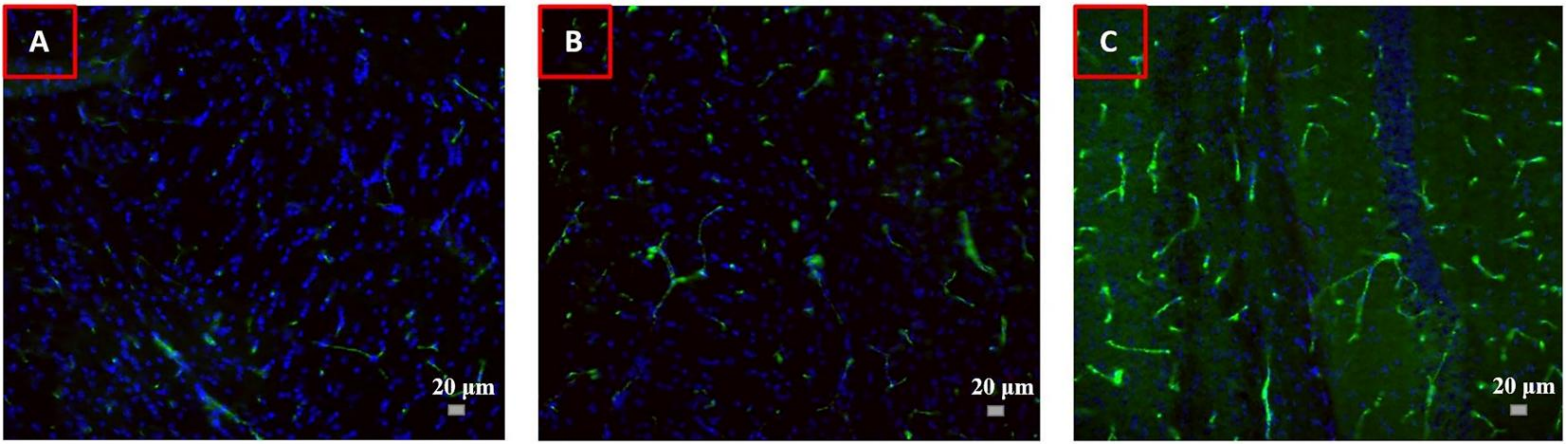
Fluorescence images of immunostained brain sections at the level of the corpus callosum for a (A) control mouse, (B) a mouse injected with EB only, and (C) a mouse treated with 30 W plus 20 μL MBs. The Fibronectin protein is stained green, and the cell nuclei are counterstained blue with DAPI (Scale bar: 20 μm)

The BBB permeability was also characterised using Fibrinogen and Fibronectin immunofluorescent staining. The mice treated with FUS plus MBs showed higher levels of the protein in all examined brain areas compared to the control mice. Images of fluorescence microscopy from the corpus callosum are presented in Figure 6, where the fibronectin is stained green, and the cell nuclei are stained blue. It seems that for the control mouse (Figure 6A) and the mouse that received EB only (Figure 6B) the protein remained in the perivascular extracellular matrix. On the contrary, in the case of the mouse treated using electrical power of 30 W and 20 μL MBs (Figure 6C), the fibronectin leakage is clearly visualised as a diffused green dye in the brain tissue.

## 4 DISCUSSION

The current study presents two robotic devices intended to facilitate preclinical research on transcranial applications of FUS in small animal models, such as mice. The specific application of the system is the FUS‐mediated BBB opening for the delivery of therapeutic drugs that are normally hampered by the BBB into the brain parenchyma.

The first version of the robotic system was developed with two piezoelectric‐actuated motion axes. The mechanical parts and FUS transducer were arranged in a single water enclosure. The rotational motion of the motors located outside the container is converted into linear motion of the respective stages inside the enclosure by Jack screw mechanisms. The system incorporates a custom made single element FUS transducer operating at a frequency of 1 MHz. Note that MBs‐enhanced pulsed FUS around 1 MHz was predominantly selected for similar applications in mice by numerous studies.^9^, ^10^, ^37^, ^38^ A specialised platform featuring four moving plates with locking levers was designed and fitted in the acoustic opening to safely immobilise rodents of different size and type above the transducer. During operation, the enclosure is filled with degassed water that serves as the coupling medium for proper beam propagation from the transducer to the mouse head.

The FUS transducer was also manufactured in‐house using a purchased piezoelectric element that was housed in a plastic case and covered by an epoxy encapsulant. The acoustic efficiency of the transducer was experimentally determined at 33% by the radiation force balance method. The produced FUS field was scanned using a hydrophone. The collected sound pressure signals were displayed on a digital oscilloscope, thus allowing assessment of the pressure field distribution. The obtained results revealed an actual location of the focal spot shifted at 7.5 cm, compared to the focal distance of 8 cm reported by the manufacturer for the element. This method also provided good indication of the size of the focal spot.

The most parts of the device were developed on a rapid prototyping machine using plastic to avoid interference with the scanner. The MRI compatibility of the developed system was assessed in a 1.5 T MRI scanner by comparing the SNR of SPGR images of an agar‐based MRI phantom obtained under different activations of the system. Regarding the positioning mechanism, noticeable SNR reduction was observed when the motion command was initiated (motor moving). Regarding activation of the FUS transducer, the image quality was getting degraded as the output power was increasing, thus resulting in some loss of detail. However, the induced SNR reductions were not considered significant. In other words, all tested activations resulted in SNR values sufficiently high for proper imaging, and thus, the efficacy of anatomical targeting and MR thermometry are not influenced. It should be though noted that since activation of the various components requires the use of electricity the system is classified as MR conditional (American Society for Testing and Materials (ASTM) standards).

The feasibility of the system in opening the BBB of small animal models using pulsed FUS in synergy with MBs was examined in WT mice. The mouse platform provided proper immobilisation of the mouse in the supine position. Targeting was though proven challenging due to the inability to directly visualise the exact location of the transducer relative to the mouse brain. However, promising results were obtained indicating successful opening of the BBB. Specifically, EB leakage in the brain parenchyma was clearly evidenced in microscopy images of brain cryosections only in the case of mice treated with FUS in synergy with MBs. It is interesting to note that the mouse treated with higher acoustic power showed higher levels of EB dye diffusing through a larger brain area. The BBB permeability was also confirmed by Fibronectin and Fibrinogen immunofluorescent staining. Again, the FUS treated mice showed higher levels of the protein in all examined brain areas, whereas for the control mouse the protein remained in the extracellular matrix.

Some issues identified during these preliminary experiments led to the development of a second improved version of the system. The first system comprises a relatively large water container that has to be filled up to the top so that the animal's head is in direct contact with the water and efficient ultrasonic propagation is achieved. However, the large water volume needed to achieve acoustic coupling makes the device heavy and less ergonomic. It was also observed that this design is prone to water leakage from the acoustic opening. Additionally, targeting the animal's brain in the laboratory setting was proven challenging due to the inability to directly visualise the transducer's location. Another identified limitation relates to the intravenous injections and administration of anaesthesia, which cannot be performed properly without removing the mouse from the device.

The second version was designed to address these issues, thus facilitating mice experiments even more. This device uses a top to bottom approach and features motion only in the vertical direction. To be more specific, the FUS transducer was integrated in a coupling cone that can be moved vertically and tightly fit the mouse head. Accordingly, the dimensions of the system were reduced considerably making the device even more compact, lightweight, and ergonomic in its use. A silicone membrane was used to seal to bottom opening of the coupling cone. The membrane unavoidably reduces the efficacy of acoustic coupling. For this reason, it was selected to be thin (0.2 mm) to minimise ultrasonic attenuation. Also, ultrasound gel was applied to displace air and maximise ultrasonic transmission. It is noted that this is a simplified device suitable for single‐shot FUS applications. A more advanced device could be developed in the future with the addition of horizontal motion stages, thus enabling sequential placement of the transducer at multiple brain locations, but at the cost of increasing size and complexity.

Additionally, the top to bottom approach allows the placement of the animal in the prone position that is much more stable, simultaneously offering better immobilisation of the mouse and visual confirmation of proper acoustic coupling. Furthermore, there is no possibility for water leakage from the cone. Finally, since the animal lies in a flat platform, there is direct access for the administration of anaesthesia, MBs and contrast agents through needles. An absorbent material was incorporated into the animal platform, thus reducing ultrasound reflections.

It is important to ensure that no bubbles obstruct the beam path. In this regard, the manual rotational mechanism of the transducer incorporated in the second version of the system is extremely useful. A simple method to remove air bubbles is to rotate the transducer at 90°, and then, once the coupling cone is filled with degassed water, rotate it back in its horizontal position. An elastic band was included in the mechanism to stabilise the transducer.

The motion accuracy of both systems was assessed following a calliper‐based methodology as previously detailed in the literature.^35^ The obtained results demonstrate that the motion error is decreasing with increasing motion step in all axes, with a maximum positioning error of about 0.1 mm for the 1‐mm step.

The single‐element spherically focussed transducer of 1 MHz that was developed in‐house was proven suitable for the specific trans‐skull application of FUS‐induced BBBD in mice, most probably due to their small skull thickness. Although very promising results were obtained, further experiments should be performed using the second version of the device, which is expected to address all the difficulties faced during the feasibility studies of the first version.

Despite the fact that the systems are mostly intended to be used in the laboratory setting, their MRI compatibility constitutes a great benefit since it allows for treatment planning and accurate targeting based on high resolution anatomical images, as well as confirmation of BBB opening by contrast agent enhanced imaging directly after treatment without moving the device from the scanner. Therefore, subsequent experiments may be benefited by treatment planning and post‐treatment BBBD assessment in the MRI setting. Note that MRI has been already employed in numerous studies mostly for assessing whether the BBB was successfully disrupted,^9^, ^38^, ^39^, ^40^ and less often for focus positioning and targeting.^39^, ^40^

It is essential to clarify that the current study focuses on the development of the two FUS robotic systems while a feasibility study on a small number of mice was only included to provide proof of concept for their intended application. Therefore, a dedicated targeting method such as the use of a stereotactic frame was not adapted. Instead, a global approach was followed, where the transducer's location was adjusted so that the FUS beam targets the skull centrally roughly focussing at the level of the hippocampus. This approach was efficient to obtain proof of successful ultrasonic coupling and disruption of the BBB. Follow up studies will focus on evaluating the second optimised version in a large number of mice accounting for specific parameters affecting the location and extent of the BBBD, as well as on assessing the ability of delivering chemotherapeutic drugs through the opened BBB.

## 5 CONCLUSIONS

Overall, the proposed devices constitute a cost‐effective and ergonomic solution for FUS mediated non‐invasive and reversible disruption of the BBB in small animal models, such as mice and rats. It should be though noted that both devices could also be used for other brain or body applications in various types of rodents, provided that their size is appropriate. The preparation of the experimental setup can be completed within a few minutes taking up minimal space. The user can remotely adjust the transducer's position and initiate sonication through a dedicated user‐friendly software. Such ergonomic devices are expected to facilitate research in the relevant field, thus accelerating clinical translation of the technology to offer an alternative therapeutic solution for neurological diseases.

## AUTHOR CONTRIBUTION

Marinos Giannakou contributed to the development of the robotic devices and draughting of the manuscript. Anastasia Antoniou contributed to the draughting of manuscript and implementation of the scientific methods. Elena Georgiou and Kleopas Kleopa contributed to the execution of the mice experiments. Christakis Damianou supervised the overall study, as well as the draughting of the manuscript.

## CONFLICT OF INTEREST

All authors declares no conflict of interest.

## Data Availability

The data that support the findings of this study are available from the corresponding author upon reasonable request.
